# Combined total internal reflection AF spectral-imaging and Raman spectroscopy for fast assessment of surgical margins during breast cancer surgery

**DOI:** 10.1364/BOE.411648

**Published:** 2021-01-19

**Authors:** Maria Giovanna Lizio, Zhiyu Liao, Dustin W. Shipp, Radu Boitor, Raluca Mihai, James S. Sharp, Matthew Russell, Hazem Khout, Emad A. Rakha, Ioan Notingher

**Affiliations:** 1 School of Physics and Astronomy, University of Nottingham, University Park, Nottingham, NG7 2RD, UK; 2 Division of Oncology, School of Medicine, University of Nottingham, Nottingham, NG5 1PB, UK; 3 Nottingham Breast Institute, Nottingham University Hospitals NHS Trust, Nottingham, NG5 1PB, UK; 4 Department of Cellular Pathology, Nottingham University Hospitals NHS Trust, Nottingham, UK

## Abstract

The standard treatment for breast cancer is surgical removal mainly through breast-conserving surgery (BCS). We developed a new technique based on auto-fluorescence (AF) spectral imaging and Raman spectroscopy for fast intraoperative assessment of excision margins in BCS. A new wide-field AF imaging unit based on total internal reflection (TIR) was combined with a Raman spectroscopy microscope equipped with a 785 nm laser. The wavelength of the AF excitation was optimized to 365 nm in order to maximize the discrimination of adipose tissue. This approach allows for the non-adipose regions of tissue, which are at a higher risk of containing a tumor, to be targeted more efficiently by the Raman spectroscopy measurements. The integrated TIR-AF-Raman was tested on small tissue samples as well as fresh wide local excisions, delivering the analysis of the entire cruciate surface of BCS specimens (5.1 × 7.6 cm^2^) in less than 45 minutes and also providing information regarding the location of the tumor in the specimen. Full automation of the instrument and selection of a faster translation stage would allow for the measurement of BCS specimens within an intraoperative time scale (20 minutes). This study demonstrates that the TIR-AF Raman microscope represents a feasible step towards the development of a technique for intraoperative assessment of large WLE within intraoperative timescales.

## 1. Introduction

Each year, it is estimated that approximately 270,000 and 55,000 new cases of breast cancer are diagnosed in the USA and UK, respectively [1,2]. Standard treatment for operable breast cancer involves surgical removal of the tumour typically using breast conserving surgery (BCS) approaches. The excision specimen is examined post-operatively and completeness of excision of the tumour is assessed through measurement of the distance between the tumour and the edge of the specimen (surgical margin). [3] It is estimated that between 10 and 20% of BCS require a second intervention (re-excision) because of positive margins involved by the tumour (tumour on ink) including small tumour foci, which can be difficult to identify intraoperatively. [4–6]

One potential solution for reducing the number of re-excisions would be to use a technique that can assess the surgical margins intra-operatively (within a reasonable time frame; typically less than 20 minutes). Several intra-operative techniques for the assessment of tumour margins have been developed and tested, such as frozen sections, ultrasound probes, radiography, fluorescence lifetime imaging and mass spectrometry. However, for each of these techniques, there are specific limitations in terms of practicality, performance or cost-effectiveness [7,8]. Intra-operative frozen section examination is a well-established method and can reduce the re-excision rates. [9] However this method is laborious, it requires highly trained staff and it can lead to false results due to sampling errors and the high rate of histological artefacts induced when processing fatty samples [7,10]. For these reasons, the evaluation of surgical margins in breast specimens is often performed on permanent section [11]. Therefore a new approach for intraoperative assessment of margin that can overcome such limitations is urgently needed.

Raman spectroscopy is a promising candidate because it provides diagnosis of breast tissue based on its chemical composition [12–16] providing quantitative analysis of the excised tissue [15,17,18]. Raman spectral imaging based on raster-scanning is slow, as it can take up to two hours to measure a 2 × 2 mm^2^ tissue sample at the spatial resolution required to detect DCIS. However, the length of time needed for the Raman analysis can be drastically reduced by the use of selective-sampling Raman spectroscopy [19–23]. Previous work has demonstrated that selective-sampling Raman spectroscopy can be successfully used to evaluate breast wide local excision specimens (WLEs) [24,25]. This method used a multimodal approach, which incorporated high-resolution confocal auto-fluorescence (AF) microscopy and Raman spectroscopy. A 405 nm excitation and 511 nm long-pass emission system was used to obtain high-resolution AF-images, and an algorithm was developed to divide the image into segments [25]. Raman spectra were then acquired from the segments with lower AF intensity, which were typically more likely to correspond to tumour, in order to establish a final label for each segment as either tumour or normal breast tissues. This approach achieved 90% sensitivity at 80% specificity and analysis was completed within 12–25 minutes for one side of the resection surface [22,25]. The main challenge in these studies was related to the size and irregular surface of the lumpectomy specimens (which limited analysis speed and accuracy) and the presence of blood. More recently, the use of Raman bands in the high-wavenumber region for fast imaging of adipose tissue combined with fingerprint Raman spectroscopy for classification of non-adipose tissue was also proposed for assessment of surgical margins in breast excisions [26].

In this study we propose an alternative approach, in which Raman spectroscopy is combined with wide-field auto-fluorescence spectral imaging in order to speed up tissue analysis. Blood contains hemoglobin which is a strong chromophore, that shows an intense absorption peak (Soret peak) at approximately 400 nm [27,28]. Thus, the use of 405 nm excitation, as used in previous reports, can result in strong absorption of the light in the tissues [29]. As a result, areas of adipose tissue that contained large quantities of blood appeared dark in the AF image (due to the absorption by the blood) and healthy adipose tissue was unnecessarily selected for the Raman analysis, increasing the overall tissue analysis time. To improve the performance of AF imaging identifying adipose tissue and thus reduce the number of Raman spectra, we investigated auto-fluorescence spectra of breast tissue using two excitation wavelengths: 405 nm (chosen to allow direct comparison with our previous work) and 365 nm (based upon a preliminary literature study) [25,30,31]. Having tested both excitation wavelengths and identified the preferred one, a device was developed and tested on small breast tissue samples to evaluate the efficiency of wide-field total internal reflection imaging to perform auto- fluorescence imaging and to identify/eliminate regions containing adipose tissue. The bespoke waveguide imaging unit was then tested on ex-vivo wide local excisions (WLEs) to optimize the TIR_AF imaging step. Once the imaging acquisition was optimized, the imaging unit was paired with, selective Raman sampling and again first tested first on small tissue samples and subsequently on fresh wide local excision. The image analysis was optimized to efficiently screen out the adipose tissue and an algorithm was developed for the semi-automated screening of WLE within 30–45 minutes.

## 2. Materials and methods

### 2.1 Tissue samples

Breast tissue samples were collected from patients undergoing breast cancer surgery at Nottingham University Hospitals NHS Trust. The study was approved by the Nottingham Health Science Biobank (NHSB, REC reference 15/NW/0685) and consent was obtained from all patients. The investigation of WLE samples was approved by the Health Research Authority (19/EM/0251).

Two types of tissue samples were used for the experiments. The first type consisted of small breast tissue samples taken from mastectomies and wide local excisions (WLE). Small tissue samples (size varying from 10 × 10 mm^2^ to 32 × 40 mm^2^ and approximately 2–10 mm thick) were used to perform fluorescence emission studies (13 samples) and collect preliminary test data (6 samples). These samples were frozen and stored at −20 °C immediately after cutting, until they were thawed for measurement. At the end of the experiments, the samples were fixed and submitted for histological processing to obtain H&E sections of the analysed surface.

WLE resections (size up to 10 × 10 cm^2^) were analysed fresh, within 1h from the surgery (16 samples). After the specimens were received from theatre they were prepared as follows: Step 1: the specimen was dipped into black ink according the standard protocol, to keep track of the margins of the specimen; Step 2: the specimen was incised from the inferior to the superior margin to expose the tumour; Step 3: the exposed surface was blotted to remove excess blood and biological fluids; Step 4 the sample was transferred to the instrument. (See Figure S1 Supplement 1).

### 2.2 Instrumentation

#### 2.2.1 Fluorescence emission spectroscopy

Fluorescence emission spectra of human breast tissues were collected at two excitation wavelengths. For 405 nm excitation, a confocal microscope (Nikon C2) with a 405 nm laser was used (Obis 405, Coherent). The emitted fluorescence light was analysed by a spectrometer equipped with a 300 lines/mm grating and silicon CCD (Andor Shamrock 303i). For 365 nm excitation, emission light from a 190 mW LED was collimated and focused using optical lens for acquiring the spectra (ThorLabs, M365L) using the same spectrometer for spectral analysis. For each sample, several locations were analysed in order to account for tissue heterogeneity. At the end of the experiments, the tissue samples were fixed, sectioned and stained to obtain the H&E sections of the tested surface. A trained pathologist identified the type of tissue (labelled as adipose tissues, stroma or tumour) at all locations where fluorescence spectra had been recorded.

#### 2.2.2 Total internal reflection (TIR) fluorescence spectral imaging

**Fig. 1.**
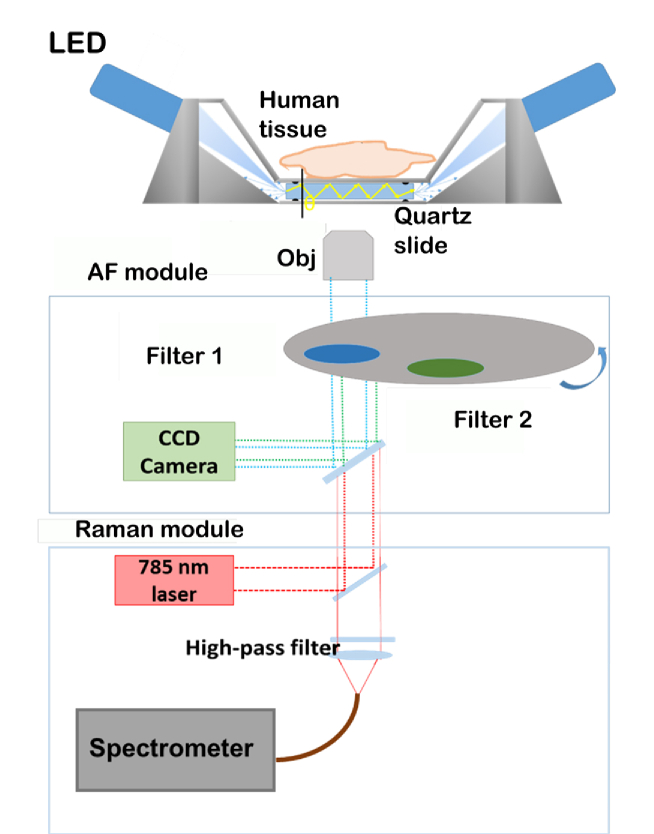
Schematic representation of the LED based waveguide TIRF system: the instrument is powered by four LEDs mounted on an aluminium holder that supports a quartz slide (1mm thick) on which human tissue is placed. The light coming out of the tissues is collected with an objective (Obj). The objective is switched between 1x, for AF module and 60x for the Raman Module. In the AF imaging system, totally internally reflected light from the LEDs is scattered at the waveguide surface (quartz slide). The incoming light with incident angle greater than 48° is trapped in the quartz slide and undergoes total internal reflection generating an evanescence field on either side of the quartz slide. The incoming light, with angles less than 48°, refracts out into the other side of the cover glass and are absorbed by the black O-ring.

A schematic of the TIR-fluorescence microscope is shown in Fig. 1. For excitation, four 365 nm wavelength LEDs (single LEDs typical power 360 mW) were mounted on an aluminium enclosure designed to fit on a microscope stage.

The LEDs were tilted at an angle of approximately 150° with respect to the stage. A cylindrical lens was used to focus the excitation light into an horizontal line in order to maximize coupling into the 1 mm thick quartz slide (5.1 × 7.6 cm^2^), on which the sample was placed. The quartz slide was mounted on a removable aluminium sandwich sealed by an O-ring on either side of the slide. The removable sandwich was built to allow better cleaning of the set-up after each experiment. Considering the mismatch between the refractive indices of quartz (n_q_=1.54) and air (n_t_=1.0003), the angle of incidence of the excitation light was larger than the critical angle (θ_c_) leading to total internal reflection and waveguiding of the excitation light in the quartz slide. The evanescent field interacts with material that is in intimate contact with the quartz slide and can be used to excite auto-fluorescence in the near surface region of the tissue.

The tissue samples were pressed into contact with the quartz slide using a 800 g weight designed for the experiments. Fluorescence images were acquired using a Nikon CFI Plan 1x/0.04 UW objective and a QImaging QIClick CCD Camera (6.45 µm pixel size). The instrument was controlled using software written in LabView (National Instruments). The microscope was equipped with two band pass filters that allowed different optical spectral regions of interested to be selected based on the results obtained from the fluorescence spectroscopy experiments. The first band pass filter selectively allowed light with wavelengths between 442 to 488 nm to pass and rejects all wavelengths outside this range (Filter 1). The second band pass filter only transmitted wavelengths between 480 and 562 nm (Filter 2). These two filters were used to obtain two separate TIR-AF images by manually switching between them during the experiments.

Once the TIR-AF images were acquired, purpose-developed software in Matlab (Mathworks) was used for processing. A virtual mask was applied to remove the background areas and isolate the area containing the tissue in the AF images. The virtual mask removes the background based on an intensity threshold that is manually adjusted by the user to ensure that the area of the images that do not contain tissue is removed [25]. Once the two AF images had been masked in this way, the image obtained from Filter 1 was divided by the image obtained from Filter 2 to obtain a ratiometric image (See TIR-AF imaging system). The resulting ratiometric images were then directly compared to H&Es of the same tissues with the support of a trained pathologist. Subsequently, different thresholds were applied to the ratiometric image to perform multi-level thresholding in order to identify ratio values that allow the isolation of stroma and tumour tissues while screening-out the majority of the adipose tissue. The ratio values selected were: 0.8<T<2 and 1<T<2 (see Results and Discussion).

#### 2.2.3 Combined TIR-AF imaging and Raman spectroscopy

The TIR-AF imaging system was combined with Raman Spectroscopy, which was implemented on the same optical microscope. An auto-focusing system for the z-axis was established so the sample was kept in focus during the Raman measurements.

Excitation for Raman spectroscopy was performed using a 785 nm CW laser (XTRA, Toptica). The laser was focused on the sample by a 60x, 0.85 NA objective that was optimized for Raman spectroscopy (River Diagnostics) and the laser power at the sample was 150 mW. Scattered light was collected through the same objective and delivered to a spectrometer (Oriel 77 200, Newport) and charge coupled device (CCD) camera (iDus DU-401-A-RR-DD, Andor) using a dichroic mirror, a longpass filter (RT785rdc and RET792lp, Chroma) and focused by a 25 mm focal length lens (AC127-025-B-ML, Thorlabs) to a 100 µm core optical fibre. Spectra were detected over the range of 600–1800 cm^−1^.

After the images were collected using the two filters and processed to obtain the ratiometric image, Raman raster scan experiments were performed by manually selecting multiple regions identified from the thresholded ratiometric TIR-AF image. The acquisition time for the Raman spectra was 3 s. Scanned areas ranged from 3 × 4 to 20 × 16 mm^2^ with 50–100 µm step-sizes.

Raman spectroscopy measurements were performed at locations independently determined by a selective sampling algorithm. The algorithm first performs threshold (T) analysis of the TIR-AF ratiometric image using the two ranges previously described (0.2<T<0.8 and 0.8<T<2). Values between 0.8 and 2 were selected for further segmentation by performing k-means cluster analysis. For samples with extended fibrosis, it was observed that the fibrosis areas were characterised by values of the intensity ratio lower than 1, therefore for those samples the range selected for further cluster analysis was restricted to 1<T<2. This analysis partitions the dataset into a number (K) of pre-defined, non-overlapping clusters in which each data point belongs to only one cluster [32]. The size of the segments (measured in pixels) was input by the user based on the size of the specimen, for small tissue sample it was fixed to 1000 px while for WLE it varied from 5000 up to 12000 px with the number of segments varying from 28 to 83 based on the size of the specimen and the area isolated by the algorithm for the clustering analysis. The segmented images were used to generate Raman sampling points. The number and location of measurement points were determined as previously described [20], with a minimum of 3 points per segment and keeping the total Raman spectral acquisition time to be approximately 15 minutes.

The acquisition time for each Raman spectra was set to 3.5 s. The processing of Raman spectra included cosmic ray removal, spectral-response correction using a NIST fluorescence standard (SRM 2241, NIST), wavenumber calibration using polystyrene as a reference, background subtraction [33], and smoothing [34] . Raman spectra with intensities larger than 3000 counts at 780 cm^−1^ were discarded (due to fluorescence interference), as were spectra with signal-to-noise ratios lower than 15.5 (signal to noise ratio was calculated as the ratio of the intensity of the at 1450 cm^−1^, CH_2_ scissors peak, divided by the standard deviation of intensities in the relatively quiet range 1370–1410 cm^−1^).

## 3. Results

Previous studies have reported that tumours have lower concentration of collagen and FAD compared to normal breast tissue, as well as a higher concentrations of both NADPH, NADH, protoporphyrin and protein when compared to stroma [30]. In contrast, lipopigments (e.g. ceroids, lipofuscin) are more abundant in adipose tissue [31]. However, auto-fluorescence intensity can vary due to fluorophore environmental factors (such as the presence of blood or increased concentration of lipopigments in the tissues due to the diet of the patient). To avoid errors due to auto-fluorescence intensity variations, we investigated the feasibility of ratiometric fluorescence imaging by calculating an image as the ratio of two fluorescence images obtained from two different spectral ranges. Ratiometic imaging has the advantage of providing a self-calibration for the signal by eliminating environmental variations, as well as other experimental conditions (e.g. variations in excitation intensity, contact between tissue and quartz window). We investigated two excitation wavelengths (405 nm and 365 nm excitation) in order to identify fluorescence spectral differences between stroma, tumour and adipose tissue, which could then be used for ratiometric imaging.

**Fig. 2.**
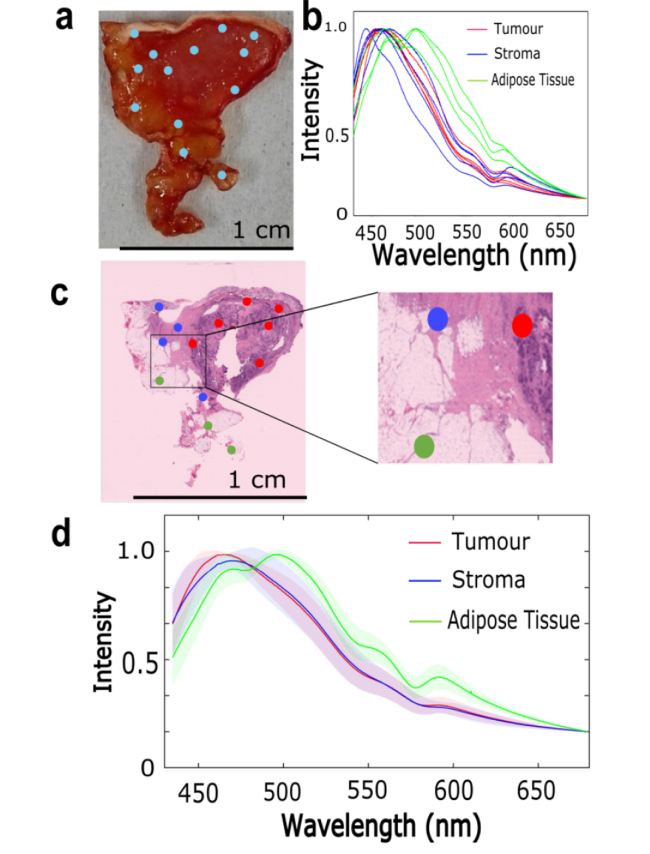
Fluorescence emission spectra of breast tissue obtained using a 365 nm excitation wavelength. a) White light photograph of a typical tissue sample, the blue dots indicate the locations from which the spectra were collected. b) Normalised fluorescence emission spectra corresponding to tumour, stroma and adipose tissue for the sample presented in a). c) H&E stained section of the sample presented in a), with the collection locations of the spectra marked by the dots colour coded based on the assigned class (tumour, stroma, adipose tissue). d) Mean fluorescence emission spectra from 10 samples (10 different patients). The shadings show the standard deviation.

Breast tissue is highly heterogeneous, therefore fluorescence spectra were collected at different locations on the sample surface, and then H&E sections were obtained to assign a class (tumour, stroma or adipose tissue) to each spectrum. This process is illustrated in Fig. 2(a)–(c) for a typical tissue sample, using the 365 nm excitation wavelength. The emission spectra corresponding to adipose tissue have two intense peaks at 475 nm and 500 nm and two smaller peaks at 550 nm and 595 nm. The positions of these peaks are consistent with data reported in the literature for healthy breast tissues and adipose breast tissue, and were assigned to vitamins, lipopigments and porphyrin, respectively [35]. In contrast, the emission spectra of tumour and stroma are very similar, showing an intense peak at approximately 460 nm that can be assigned to NADPH and NADH [30,35]. The mean fluorescence emission spectra corresponding to 365 nm excitation that were collected from ten samples obtained from ten patients are presented in Fig. 2(d). The results show that the 550 nm peak in the spectra of adipose tissue, typical of lipogiments, is consistent among the different samples studied and can be clearly resolved from other spectral features. However, the results show that no significant differences exist between the emission spectra of stroma and tumour, when 365 nm light is used for excitation.

**Fig. 3.**
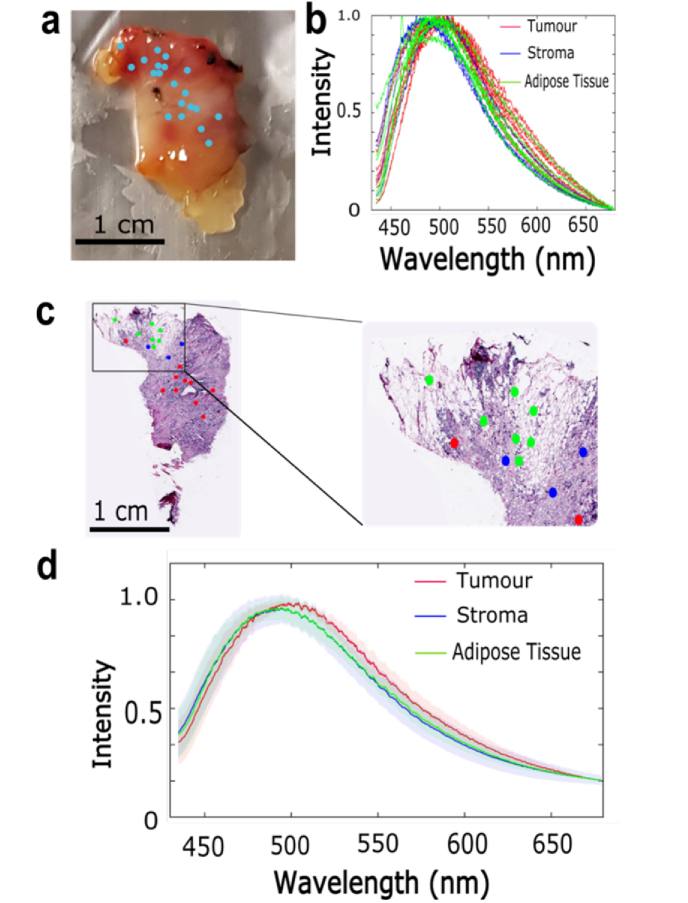
Fluorescence emission spectra of breast tissue obtained using a 405 nm excitation wavelength. a) White light photograph of a typical tissue sample, the blue dots correspond to the locations from which spectra were collected. b) Normalised fluorescence emission spectra corresponding to tumour, stroma and adipose tissue for the sample presented in a). c) H&E stained section of the sample presented in a), the dots correspond to the location from where the spectra were collected and are represented colour coded based on the assigned class (tumour, stroma, adipose tissue). d) Mean fluorescence emission spectra from 6 samples (6 different patients). The shadings show the standard deviation.

The fluorescence emission spectra of breast tissue measured using 405 nm excitation wavelength are presented in Fig. 3. Similar to Fig. 2, Fig. 3(a)–(c) show the results for a typical breast tissue containing tumour, stroma and adipose tissue. However, compared to the spectra obtained at 365 nm excitation, the results in Fig. 3(b) show only minor spectral differences between the tissue types. The spectra measured at locations corresponding to adipose tissue have an intense peak at ∼500 nm while a similar peak can be observed at ∼490 nm for tumour and stroma. The mean spectra obtained from six samples (six patients) confirmed these findings (Fig. 3(d)). Comparing the results of the fluorescence emission spectra obtained with 365 nm and 405 nm excitation wavelengths, it was concluded that 365 nm wavelength provided larger spectral differences between adipose tissue and the other tissue types. The 365nm excitation wavelength was therefore selected for use in TIR-AF imaging.

**Fig. 4.**
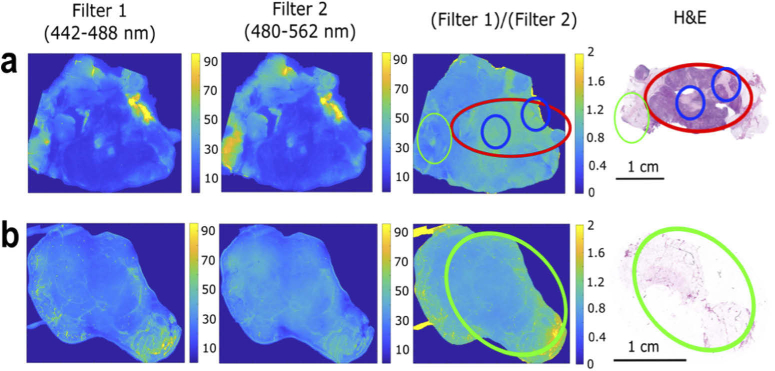
TIR-AF imaging of typical breast tissue samples. a - Sample containing tumour, stroma and adipose tissue. b- Sample containing only adipose tissue. The TIR-AF images were collected using Filter 1 (442–488 nm) and, Filter 2 (480–562 nm), the ratiometic images were calculated dividing image from Filter 1 by the image from Filter 2. The red circle represents the tumour area, while green and blue circles highlight the adipose tissue and stroma. The adjacent H&E stained sections (included for comparison). Additional information in supplemental document Figure S2.

Figure 4 compares the TIR-AF images for typical breast tissue samples with the adjacent H&E stained sections. Fluorescence emission images obtained with the Filter 1 (442 to 488nm) and Filter 2 (480 to 562nm) were acquired using the TIR-AF imaging unit (shown in Fig. 1), using a 365 nm excitation wavelength. After optimisation of the main microscope and camera parameters (camera gain, resolution, speed of the microscope translation stage), TIR-AF images of an area of 2 × 4 cm^2^ of tissue were acquired in 4 minutes (1.6 minutes for actual image acquisition and 2.4 minutes required for scanning the sample using the microscope translation stage, see Supplement 1 section 3).

The sample in Fig. 4(a) contained both normal breast tissue (adipose tissue and stroma) and tumour, while the sample in Fig. 4(b) consisted only of normal breast tissue. In the individual images recorded with each filter, Fig. 4 shows that adipose tissue has higher fluorescence intensity compared to stroma and tumour. A greater consistency was obtained between samples from different patients when calculating ratiometric images (Fig. 4, Filter1/Filter2). The majority of the adipose tissue areas are characterized by ratio values that are typically smaller than 1, while tumour and stroma have values in the range 1–2. These results are in agreement with the emission intensity spectra in Fig. 2. The adipose tissue has an intense band corresponding to Filter 2 (480 to 562nm) and therefore it is expected that the ratio values would be lower compared to tumour and stroma. Although regions of adipose tissue in Fig. 4(b) appear to have ratio values higher than 1, it is important to note here that the aim of the TIR-AF imaging is to exclude as much area of adipose tissue as possible, for the subsequent Raman spectroscopy measurements. This means that although some areas of adipose tissue are incorrectly identified as tumour or stroma, the area of the tissue that needs to be sampled using Raman measurements are greatly reduced. However, it is worth stressing at this point that although adipose tissue was occasionally misidentified as other tissue types using the TIR-AF techniques, tumour and stroma were never misidentified as adipose tissue.

In order to identify consistent values in the ratiometric image that could allow for an automated detection of adipose tissue, we performed multi-level thresholding to divide the ratiometric images in to multiple regions based on different ratio values. The different regions isolated with different ratio value ranges were plotted in separate images and compared with the H&E (supplemental document Figure S3). After the multi-level thresholding had been applied to all the fluorescence ratiometric images (4 small tissue samples and 3 WLE), it was observed that the adipose tissue consistently fell within ratio range 0.2 - 0.8 of the ratiometric images. All the other tissue types (stroma, inflamed stroma and tumour) were consistently isolated by the ratio range 0.8 - 2. Therefore, an algorithm was developed to apply a threshold value of T=0.8 to the ratiometric images, and regions with values smaller than this threshold were labelled as “adipose tissue”. This allows for the quick screening of adipose tissue that should also be insensitive to inter-patient variations or tissue-slide contact. It is important to note here that when setting the threshold, it is important that it is not set too low and no tumour is incorrectly classified as adipose tissue and then excluded from Raman spectroscopy. The alternative is preferable, for example areas of adipose tissue having ratio values above the threshold, in which case they will be scanned by Raman spectroscopy.

**Fig. 5.**
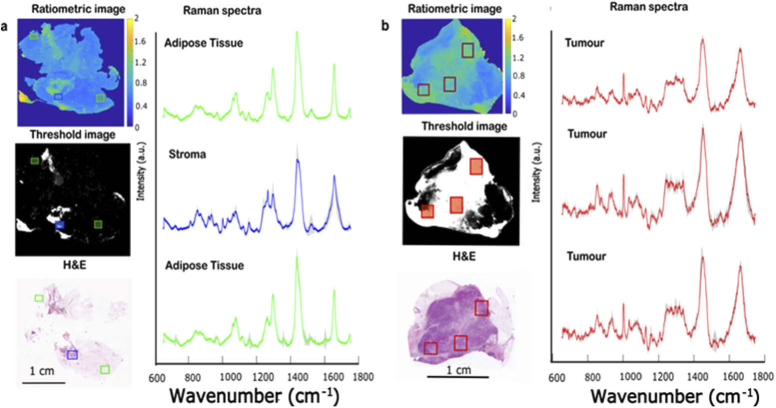
a- Breast tissue sample containing adipose tissue and stroma. The green and blue boxes indicate the location selected for the Raman measurement. The figure reports: ratiometric AF image, threshold image (T<0.8), H&E staining and the average of Raman spectra acquired from the area corresponding to the areas highlighted by the squares: adipose tissue (green spectra) and region of the tissues containing stroma (blue spectrum). The grey shadow overlaid with the spectra represents the standard deviation. The spectra were overlaid and shifted vertically for clarity. b. Breast tissue sample containing adipose tissue, stroma and tumour. The red boxes indicate the location selected for the Raman measurement. The image reports: ratiometric AF image, threshold image (0.8<T<2), H&E staining and the average of the Raman spectra acquired from the different locations. The spectra were presented as in sample a. The H&Es were obtained from the mirror image tissue so it does not overlap fully with the tissues image reported.

To test the consistency of the T=0.8 threshold, we recorded TIR-AF ratiometric images from new breast tissue samples and we compared Raman spectra recorded at locations of tissue corresponding to ratio values smaller and larger than the threshold value. This was achieved by manually moving the sample to the selected regions after the analysis of the TIR-AF image while the sample was still on the microscope stage. This test would overcome potential inconsistencies when comparing the TIR-AF and H&E images. This is because, although H&E staining was the standard of reference, it was not possible to overlap the H&E images and the TIR-AF images with sufficient accuracy, because the tissue processing required for H&E staining altered the overall shape of the tissue. On the other hand, the differences between the Raman spectra of adipose tissue, stroma and tumour are well established [14,36]. Fig. 5(a) and (b) show the ratiometric TIR-AF image and the segmented images using T=0.8 for two breast tissue sample obtained from two different patients. Sample a) contained stroma and adipose tissue; sample b) contains tumour stroma and adipose tissue. For sample a) Raman spectra collected from the areas of the tissue corresponding to ratio values between 0.2 and 0.8 (below threshold T=0.8) showed Raman spectra typical of adipose tissues, as characterised by a peak 1655 cm^−1^, (assigned to C = C in lipids), an intense peak at 1441 cm^−1^, (assigned to CH_2_ deformation of the lipids), two peaks at 1260 cm^−1^ and 1275 cm^−1^ (assigned to = CH deformations) and the complex peaks between 1080–1095 cm^−1^(assigned to skeletal vibration and PO_2_ vibrations). [14,16,24,37] On the other hand, the Raman spectra measured from the regions within ratio values above the threshold T=0.8 (and smaller than 2) presented typical Raman spectral features associated with stromal tissue, such as bands assigned to collagen peaks at 860 cm^−1^ and 938 cm^−1^ and the phenylalanine peak at 1004 cm^−1^ [14,24,25,36,38]. Raman analysis of the tissues is supported by the H&E staining that indicate large areas of adipose tissue and small regions of stroma.

For the sample in Figure5b, which contains a large area of tumour surrounded by adipose tissues, the spectra collected from areas within the 0.8 and 2 range (above threshold T=0.8) have similar bands to Raman spectra of breast tumour reported previously. [22,34,35,36,37] In particular, the peak at ∼1004 cm^−1^ associated with the phenylalanine of the proteins (which are largely abundant in tumour cells compared to healthy cells), the peak at 1655 cm^−1^ in the amide I and the peaks corresponding to collagen at 860 cm^−1^, 938 cm^−1^, which are less intense than the band at 1004 cm^−1^, have been previously reported as typical of tumour tissue Raman spectra. The corresponding area on the H&E staining confirms that the area analysed by Raman corresponds to large areas of tumour.

Next, we investigated the feasibility of using the segmented ratiometric TIR-AF images to generate sampling points for Raman spectroscopy in order to speed up the diagnosis of wide local excision specimens. Here, the tissue surface exposed tissue during this cruciation was used because it provides a better comparison with the H&E images (H&E of the surface of the lumpectomy specimens cannot be obtained with the standard histology process). Furthermore, by analysing the cruciate surface we obtain, with one single image, a snapshot of the position of the tumor and the relative distance from the edge of the tissue which is what is measured as margins.

**Fig. 6.**
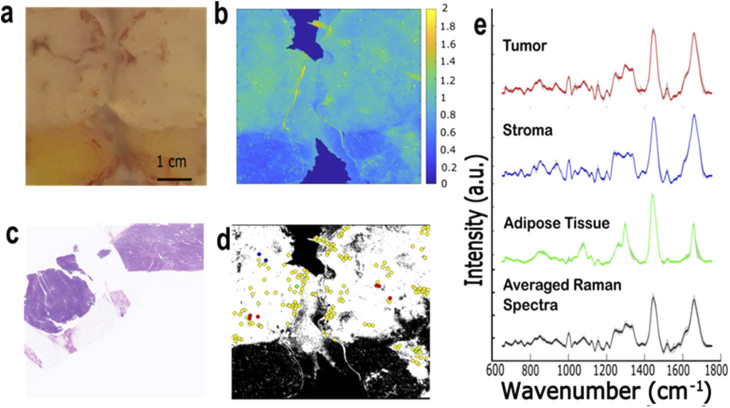
Combined TIR-AF and Raman spectroscopy analysis of a WLE specimen containing a phyllodes tumour. a- wide field (white light) image. B- ratiometric TIR-AF image. C- H&E stained section. d- threshold image (0.8<T<2) with the sampling locations for the Raman spectroscopy overlaid as yellow dots. Few locations have been highlighted and the average Raman spectra are reported in e. The two blue dots correspond to stroma, 6 red dots correspond to tumour and the two green dots correspond to adipose tissue. e- Raman spectra (processed) from the corresponding locations reported in red, blue and green while in black is the average of all the Raman spectra. The grey shadowing represents the standard deviation.

To obtain a diagnosis of a tissue sample, the segments in the ratiometric image with ratio values smaller that a threshold T=0.8 were labelled as “adipose tissue” and excluded from any further analysis. Figure 6 shows an example of multiodal analysis using TIR-AF ratiometric approach to guide Raman spectroscopy analysis of a WLE containing a phylloydes tumour, a form of tumour for which surgical removal is the preferred strategy [39]. As confirmed by the H&E staining, TIR-AF identified the adipose tissue and efficiently focused the Raman spectra at the tissue location corresponding to the tumour (a total of 168 sampling points). Figure 6 shows the processed Raman spectra from different locations as well as the average of Raman spectra.

The TIR-AF image acquisition for the WLE was set to 12 minutes while the integration time for the individual Raman measurements was fixed to 3.5 s, leading to ∼45 minutes overall analysis for the cruciate surface. Comparison of the H&E with the threshold AF image and Raman analysis, confirmed that the adipose tissue was efficiently screened out by the imaging step and the area containing tumour was correctly identified by the Raman analysis.

## 4. Discussion

Previous studies showed that the combination of auto-fluorescence imaging and Raman spectroscopy can be a powerful approach for analysis of breast tissue samples [22,40,41]. However, the application for breast surgery has been limited because of the nature, as well as the size of the samples. Compared to confocal fluorescence imaging using the 405 nm laser previously reported [25], the TIR-AF takes advantage of the characteristic fluorescence spectra of adipose tissue that enables its rapid identification. Compared to fluorescence intensity, the ratiometric approach overcomes potential variations in fluorescence intensity caused by the presence of blood, interpatient variation, spatial variations in excitation intensity and the quality of tissue contact with the quartz slide. The confocal set-up reported previously [25] was not efficient in screening out adipose tissue. Therefore, even if the total analysis time would be similar, screening out the adipose tissue by optimized excitation wavelength would allow a more efficient targeting of the “higher-risk” non-adipose areas of the tissue, providing superior diagnosis.

As typical breast resection specimens contain large areas of adipose tissue, which is efficiently identified by TIR-AF, only a small fraction of tissue surface needs to be subsequently analysed by Raman spectroscopy. Even if TIR-AF cannot distinguish between tumour and stromal tissues, the efficient screening of the adipose tissue allows to focus the Raman spectroscopy measurements on stroma and tumour regions only where it can efficiently detect more subtle molecular differences between the tissues. More efficient exclusion of adipose tissue enabled an 8-fold increase in the acquisition time for each Raman spectrum and an increase in the signal to noise ratio.

Other important advantages of the TIR-AF system when compared to confocal AF are its simplicity and low cost. The TIR-AF system consists of an aluminium unit, quartz slide, LEDs for excitation and CMOS camera for image acquisition. The system is easy to align and operate as it does not require scanning elements. The use of the waveguide also makes the imaging system versatile, as LEDs come in a wide range of excitation wavelengths and the same waveguide unit can be easily adapted to investigate other types of tumour. The use of LEDs as the power source to illuminate a waveguide has the advantage of limiting the variation in intensity of the evanescent field that interacts with the sample compared to lasers, because the light source illuminates the entire surface simultaneously [42].

The current experimental set-up, adapted from a pre-existing microscope, allowed us to successfully measure large specimens within 45 minutes. TIR-AF images were acquired in 15 minutes (of which 13 minutes were used by the translational stage to move and to manually switch between the two filters), Raman analysis was performed in approximately 14 minutes and the rest of the time was used by the user to manually export the images and feed them into the algorithm to obtain the sampling points. The optimized discrimination of the adipose tissue using TIR –AF imaging, which better targeted high-risk areas allow to reduce the number of Raman spectra that needed to be acquired which allowed for longer acquisition time (3.5s). The longer acquisition time provides better SNR and thus better classification of the tissue structures. Full automatization of the process (switch between AF and Raman), as well as more efficient equipment (faster translation stage), will reduce the time for the screening of the specimen even further, down to an estimated 20 minutes (5 minutes to obtain the ratiometric image and 15 minutes for the Raman measurements). Moreover, by performing the analysis on the dissected (cruciated) specimen surface rather than from the outer excision surface, as previously reported in the literature, the procedure allowed for the acquisition of a snapshot of the tumour as well as the surplus healthy tissue surrounding it, as removed during surgery. This would allow for the measurement of the distance between the edge of the specimen and the tumour (surgical margins) at the end of the experiment (this could not be done by scanning the external surface of an intact specimen given the limited penetration of both AF-TIR and Raman spectroscopy). Although measuring the cruciated specimen would have the same limitations in terms of sampling errors as standard histology, it may provide faster analysis compared to scanning the entire external surface of the tissue specimen (multiple tissue slices could analysed in parallel) and would allow direct comparison to histology (obtaining histology section of the external surface of the entire tissue specimen is very difficult and impractical). As the focus of this study was on establishing the TIR-AF Raman system, we included only larger breast tumours (invasive carcinomas) in order to avoid errors when comparing the TIR-AF images to the reference histology section. Future studies will need to include smaller tumours, such as ductal carcinomas in-situ (DCIS), which account for the majority of cases of positive margins.

## Notes

See Supplement 1 for supporting content.
