# Supplementary material for: Combined total internal reflection AF spectral-imaging and Raman spectroscopy for fast assessment of surgical margins during breast cancer surgery
Source: Biomed Opt Express. 2021 Jan 19;12(2):940–54. doi: 10.1364/BOE.411648 (PMC7901337; doi:10.1364/BOE.411648)
Supplement: Supplementary file 1 [file boe-12-2-940-s001.pdf]

Supplemental Document

Biomedical Optics EXPRESS

Combined total internal reflection AF spectral-imaging and Raman spectroscopy for fast assessment of surgical margins during breast cancer surgery: supplement

MARIA GIOVANNA LIZIO,[1] ZHIYU LIAO,[1] DUSTIN W. SHIPP,[1] RADU BOITOR,[1] RALUCA MIHAI,[2] JAMES S. SHARP,[1] MATTHEW RUSSELL,[4] HAZEM KHOUT,[3] EMAD A. RAKHA,[2] AND IOAN NOTINGHER[1,*]

[1]*School of Physics and Astronomy, University of Nottingham, University Park, Nottingham, NG7 2RD, UK*
[2]*Division of Oncology, School of Medicine, University of Nottingham, Nottingham, NG5 1PB, UK*
[3]*Nottingham Breast Institute, Nottingham University Hospitals NHS Trust, Nottingham, NG5 1PB, UK*
[4]*Department of Cellular Pathology, Nottingham University Hospitals NHS Trust, Nottingham, UK*
[*]*Ioan.Notingher@nottingham.ac.uk*

This supplement published with The Optical Society on 19 January 2021 by The Authors under the terms of the Creative Commons Attribution 4.0 License in the format provided by the authors and unedited. Further distribution of this work must maintain attribution to the author(s) and the published article's title, journal citation, and DOI.

Supplement DOI: https://doi.org/10.6084/m9.figshare.13299893

Parent Article DOI: https://doi.org/10.1364/BOE.411648

Combined total internal reflection AF spectral-imaging and Raman spectroscopy for fast assessment of surgical margins during breast cancer surgery

Maria Giovanna Lizio [1], Zhiyu Liao [1], Dustin W. Shipp [1], Radu Boitor[1], Raluca Mihai[2], James S. Sharp [1], Matthew Russel[2], Hazem Khout[3], Emad A. Rakha[2], Ioan Notingher [1],*

[1] School of Physics and Astronomy, University of Nottingham, University Park, Nottingham, NG7 2RD, UK
[2] Division of Oncology, School of Medicine, University of Nottingham, Nottingham, NG5 1PB, UK.
[3] Nottingham Breast Institute, Nottingham University Hospitals NHS Trust, Nottingham, NG5 1PB, UKPublications Department, The Optical Society, 2010 Massachusetts Avenue NW, Washington, DC 20036, USA

**Ioan.Notingher@nottingham.ac.uk*

1. **Wide Local Excision sample preparation**

Fig.S1 reports an example of preparation of wide local excision specimen.

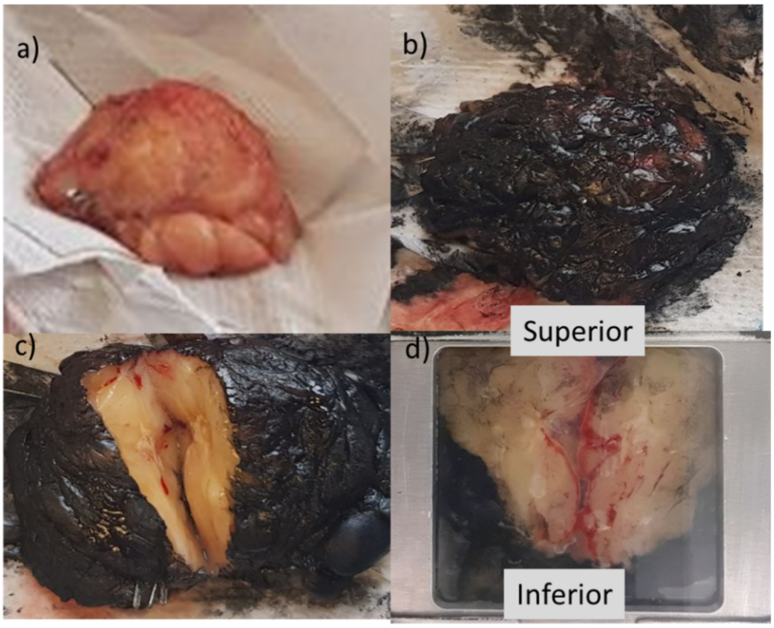

Fig. S1. Picture showing the different steps to prepare the samples for the experiment. a- The sample is dipped in ethanol to dry some of the adipose tissue. b-The sample is dipped in black ink (to recognize the edge of the tissue in the H&E slide). c-The WLE is cut from the inferior to the superior margin and d- the sample is placed onto the waveguide unit to measure the inside of the specimen.

2. Total internal reflection imaging system

The light generated by powering the LEDs will be scattered at the surface of the waveguide at different angles. For angles θc greater than 48°, which is the critical angle value of the quartz/air system calculated using Snell's law, the light will be total internally reflected within the quartz slide generating an electromagnetic field known as an evanescent wave, on the outer surfaces of the quartz. The evanescent wave has the same wavelength (λ) as that the light that is totally internally reflected, in this case 365 nm, and the energy of the wave concentrate on the proximity of its source, in our case at the quartz/air interface. The intensity of the evanescent wave (I) decays exponentially with the increase of the distance from the interface where the wave originates, with the characteristic exponential decay depth d, therefore it can excite only the fluorophores in close proximity of the interface, typically within 200 nm from the surface of the quartz. The tissues that are within the penetration depth of the evanescent wave will interact with the light of the evanescence wave. Part of the light will be scattered and part of the light will be absorbed by the fluorophores, therefore to collect AF images we used two filters that blocked the scattered light and collected only small ranges of the emitted fluorescence light, Filter 1 (442 to 488 nm) and Filter 2 (480 to 562 nm).

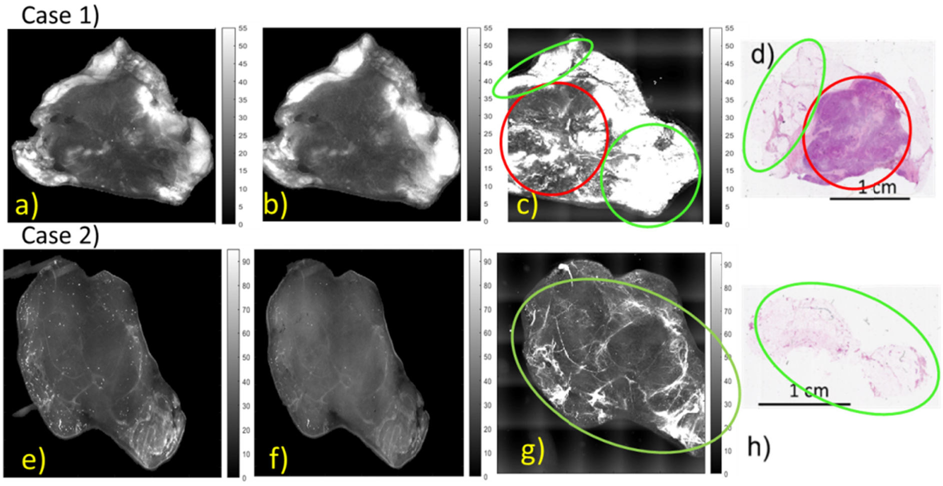

Fig. S2. a, e- TIR-AF image obtained using the waveguide set-up with 365 nm excitation from Filter 1 (442-488) for two different samples. b,f- TIR-AF image from Filter 2 (480-562). c, g- confocal auto fluorescence image using 405 nm excitation. d,h- H&E stain. Case 1- breast tissue sample containing a mixture of stroma, adipose tissue and tumour. Case 2- breast tissue sample contains primarily adipose tissue. The sample has been collected from two different patients. . The red circle highlights areas containing tumour, the green circle areas containing adipose tissue.

3. Experimental set-up for TIR AF images

The CCD camera tested for the experiments allowed the acquisition of images of 1392x1040 pixels. The size of the area scanned with one acquisition depends upon the magnification of the objective used for the experiments. For instance, the 2x magnification objective will scan an area of 4.5 x 3.4 mm^2 while the 1x objective scan an area of 9x7 mm^2. Therefore, to obtain the image of a whole tissue multiple image, "tiles", need to be acquired and stitched together with the use of software to obtain the image of the whole specimen (5.1 x 7.6 cm^2). The time necessary to acquire an image will depend upon the time necessary to acquire each tile (acquisition time), the time for the stage to move to a new location to cover the whole sample area, the number of tiles that need to be acquired and the software timing for stitching the tiles and producing the final image. We found that the best experimental condition was using the 1x magnification objective and 700 ms acquisition time (per tile) for small tissue samples cut out of mastectomies, and 1 s per tile for fresh wide local excisions. The 1x objective provided sufficient magnification to discriminate between adipose tissue and other type of structures yet allowed the image of a whole tissue to be obtained in few minutes. For instance, to acquire an image of an area of 2 x 4 cm^2 using the 1x objective and 700 ms acquisition it took a total of 2 minutes. Fig.S2 shows the TIR-AF images for breast tissue samples acquired using Filter 1 (443 to 488 nm) and Filter 2 (480 to 562 nm), the confocal images obtained with the 405 nm excitation for the same samples and the H&E stain. The comparison between the image collected with the waveguide (365 nm excitation) and the confocal (405 nm excitation) showed that TIR-AF images acquired using the waveguide set-up has good signal-to-noise. Currently, the motorised stage on the microscope is a limiting factor with 2 seconds necessary to move the stage between locations. Therefore, to collect an image composed of 70 tiles using an integration time of 700 ms per tile it took 4 minutes to acquire an image of the entire tissue. Considering the 2s reaction of the stage, about 2.4 minutes were used by the stage and 1.6 minutes where used to acquire the tiles and for the home built software to stitch them together to produce the final image. As expected the images showed the AF images obtained using waveguide TIR system are less influenced by blood when compared to the confocal microscope (Fig.S2). In particular, for Case 1 the sample contains tumour, stroma and adipose tissue, and both TIR-AF images (Filter 1 and Filter 2) show the presence of two main tissues types which is in line with the emission spectra results which suggested that the 365 nm excitation can efficiently discriminate adipose tissue from other type of structure but not tumour from stroma. The confocal image also showed differences between adipose tissues and other type of structure. By contrast, for Case 2, which contains only adipose tissue, the TIR-AF images show uniformity in the intensity of the tissues while the confocal images appear darker and non- uniform compared to the TIR-AF images.

4. Ratiometric image thresholding

The ratiometric image was analysed using a multi-level thresholding in order to identify ratio values to identify the adipose tissue. The different threshold were split to obtain 8 images, one for each range investigated and the threshold images were compared to the H&E staining of the tissue (Fig. S3).

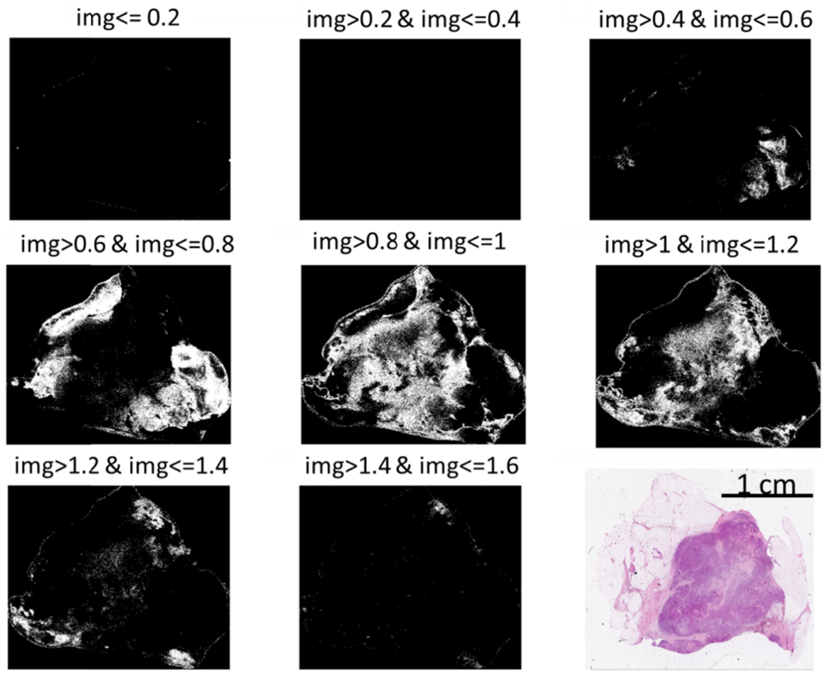

Fig. S3. Images display the area of the ratiometric image comprised in set range of the ratio values. The range set were: values of the ratio below 0.2 (img<=0.2). Ratio value between 0.2 and 0.4 (img>0.2&&img<=0.4). Ratio value between 0.2 and 0.4 (img>0.2&&img<=0.4). Ratio value between 0.6 and 0.8 (img>0.6 &&img<=0.8). Ratio value between 0.8 and 1 (img>0.8 &&img<=1). Ratio value between 1 and 1.2 (img>1 &&img<=1.2). Ratio value between 1.2 and 1.4 (img>1.2 &&img<=1.4). Ratio value between 1.4 and 1.6 (img>1.4 &&img<=1.6). The last image displays the H&E stain of the tissue.
